# Hydrophobic Silicon Quantum Dots for Potential Imaging of Tear Film Lipid Layer

**DOI:** 10.3390/nano15070552

**Published:** 2025-04-04

**Authors:** Sidra Sarwat, Fiona Stapleton, Mark D. P. Willcox, Peter B. O’Mara, Maitreyee Roy

**Affiliations:** 1 School of Optometry and Vision Science, University of New South Wales (UNSW), Sydney 2052, Australia; s.sarwat@unsw.edu.au (S.S.); f.stapleton@unsw.edu.au (F.S.); m.willcox@unsw.edu.au (M.D.P.W.); 2 Electron Microscope Unit, School of Chemistry, Mark Wainwright Analytical Centre, University of New South Wales (UNSW), Sydney 2052, Australia; peter.omara94@gmail.com

**Keywords:** tear film lipid layer, dry eye disease, hydrophobic quantum dots, fluorescence imaging

## Abstract

The tear film, consisting of the aqueous and lipid layers, maintains the homeostasis of the ocular surface; therefore, when disturbed, it can cause dry eye, which affects millions of people worldwide. Understanding the dynamics of the tear film layers is essential for developing efficient drug delivery systems for dry eye disease. Quantum dots (QDs) offer the potential for real-time monitoring of tear film and evaluating its dynamics. Hydrophilic silicon QDs (Si-QDs) have already been optimised to image the aqueous layer of the tear film. This study was conducted to optimise hydrophobic Si-QDs to image the lipid layer of the tear film. Si-QDs were synthesised in solution and characterised by transmission electron microscope and spectrofluorophotometry. The fluorescence emission of Si-QDs was monitored in vitro when mixed with artificial tears. The cytotoxicity was assessed in cultured human corneal epithelial cells using an MTT assay following 24 h of exposure. Si-QDs were 2.65 ± 0.35 nm in size and were non-toxic at <16 µg/mL. Si-QDs emitted stable green fluorescence for 20 min but demonstrated aggregation at higher concentrations. These findings highlight the potential of hydrophobic Si-QDs as a biomarker for the real-time imaging of the tear film lipid layer. However, further research on surface functionalisation and preclinical evaluations are recommended for enhanced solubility and biocompatibility in the ocular surface.

## 1. Introduction

Dry eye disease (DED) is a multifactorial disease of the ocular surface characterised by a loss of homeostasis of the tear film [1]. DED is accompanied by ocular symptoms in which tear film instability and hyperosmolarity, ocular surface inflammation and damage, and neurosensory abnormalities play etiological roles [1,2]. It is among the most prevalent ocular conditions for people seeking eye care [3,4,5]. The prevalence of dry eyes ranges from 5 to 50% [6], affecting the quality of vision and life [7]. An altered/deficient tear film is important in the pathophysiology of the DED [8].

The tear film is a complex dynamic fluid that covers the anterior ocular surface [1]. It consists of an outer lipid layer and an underlying muco-aqueous layer, which play an important role in protecting and lubricating the ocular surface [5]. Any change/lack in the aqueous or lipid layer of the tear film can lead to aqueous-deficient or evaporative DED, respectively. The prevalence of evaporative DED is significantly higher than that of aqueous-deficient DED [9]. Any change in the composition or production of tear film lipids may lead to the development of evaporative DED [2].

A range of diagnostic tests and assessments are utilised to evaluate the presence and severity of evaporative DED. These tests focus on tear film stability, lipid layer integrity, and ocular surface health [10]. One of the primary diagnostic tools for evaporative DED is measuring tear film break-up time (TBUT), which assesses the stability of the tear film [11]. However, this test does not accurately assess tear dynamics over time. In addition, ocular surface staining with fluorescein or lissamine green is used to examine the cornea, conjunctiva, and eyelid margins. These dyes disrupt the tear film during the examination [12] and are susceptible to autofluorescence and photobleaching [13]. The Schirmer test assesses tear production by inserting a strip of paper into the lower conjunctival sac [14]. Interferometry [15] and optical coherence tomography [16] have been commonly used in clinical practice to assess lipid layer thickness; however, the impact of dynamic changes and tear film stability is still not well understood [5].

The tear film is subject to dynamic interfacial interactions between its layers [17] and continuous blinking [12]. The interfacial properties impede the investigation of the phase dynamics of the tear film [12]. The information gathered from diagnostics tests does not provide insight into the tear film’s layers [12]. Therefore, the knowledge of the interfacial dynamics of individual tear film layers remains inadequate [5,18]. The simultaneous imaging of tear film layers with high-contrast biomarker may address this issue and provide more insight into tear film dynamics [12].

Quantum dots (QDs) offer a promising alternative to traditional imaging techniques and organic dyes and provide strong fluorescence for tear film bioimaging due to their excitation range of around 500 nm [13]. These nano-sized particles (2–20 nm) emit stable, bright fluorescence with reduced photobleaching, making them ideal for bioimaging [19,20]. While cadmium-based QDs are widely used, their toxicity raises concerns, especially for ocular applications [21]. However, silicon QDs (Si-QDs) present a biocompatible option with flexible surface modification, producing either a hydrophilic or hydrophobic charge, making them suitable for imaging tear film layers [22]. Silicon nanomaterials in the 50–150 nm range are compatible with human corneal cells, highlighting their potential as safer alternatives for ocular surface bioimaging [22,23,24,25,26,27].

High-contrast biological biomarkers for the precise labelling of tear film layers may enhance the knowledge of tear film dynamics [12]. In our previous study, amine-terminated hydrophilic Si-QDs have been optimised for bioimaging tear film aqueous layer [22]. However, the transition to hydrophobic systems in this study was required to image the lipid layer of the tear film, which plays a crucial role in maintaining tear film stability and preventing evaporation. Hydrophobic QDs offer several advantages, including improved compatibility with lipid-rich environments, enhanced stability at the air–liquid interface, and stronger interactions with the tear film’s outermost layer. This shift addresses the limitations observed in aqueous systems, where hydrophilic QDs primarily interact with the aqueous layer, making it challenging to study lipid-layer dynamics. Using hydrophobic QDs, we aim to expand the potential of QD-based imaging for a more comprehensive analysis of tear film structure and function.

This study presents the optimisation of hydrophobic scandium-doped Si-QDs (Sc-Si-QDs), which may eventually serve as a biomarker for imaging the tear film lipid layer.

## 2. Results

### 2.1. Characteristics of Si-QDs

**Figure 1:**
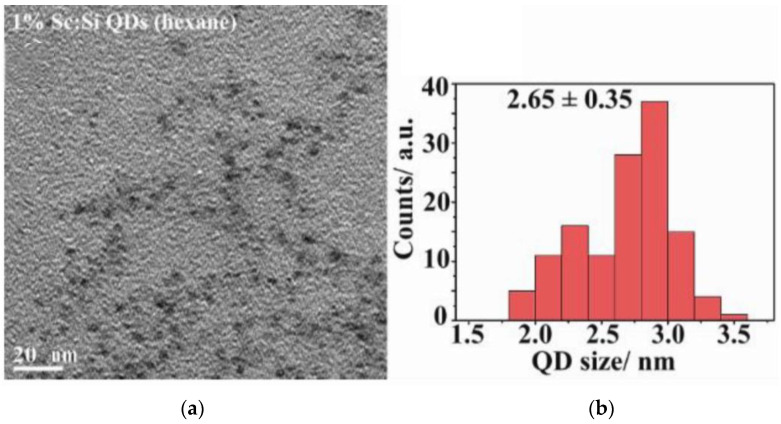
(**a**) TEM (Transmission electron microscopy) image of Si-QDs; (**b**) size distribution of Si-QDs.

Si-QDs were 2.65 nm (±0.35) in size and had spherical morphology (Figure 1).

**Figure 2:**
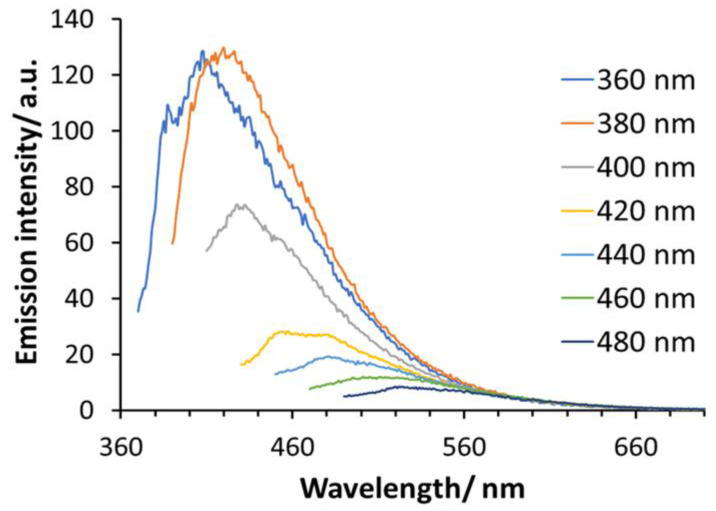
The photoluminescence emission spectra of Si-QDs at different excitation wavelengths (400–520 nm).

Figure 2 shows the photoluminescence emission spectra of Si-QDs at various excitation wavelengths (360–480 nm). The *y*-axis represents the arbitrary units of photoluminescence intensity of emission wavelengths. Si-QDs were excited remarkedly by 360, 380 and 400 nm. The emission peak (130 RFU) transitioned from 400 nm with a narrow emission bandwidth within the visible light spectrum, to 570 nm. The intensity of emitted light diminished at excitation wavelengths of 500 nm and onwards. Si-QDs were prepared using the same synthesis method; their photoluminescence quantum yield was 14.5%, and full width at half maximum was 90 nm [28,29].

### 2.2. Cytotoxicity of Si-QDs

**Figure 3:**
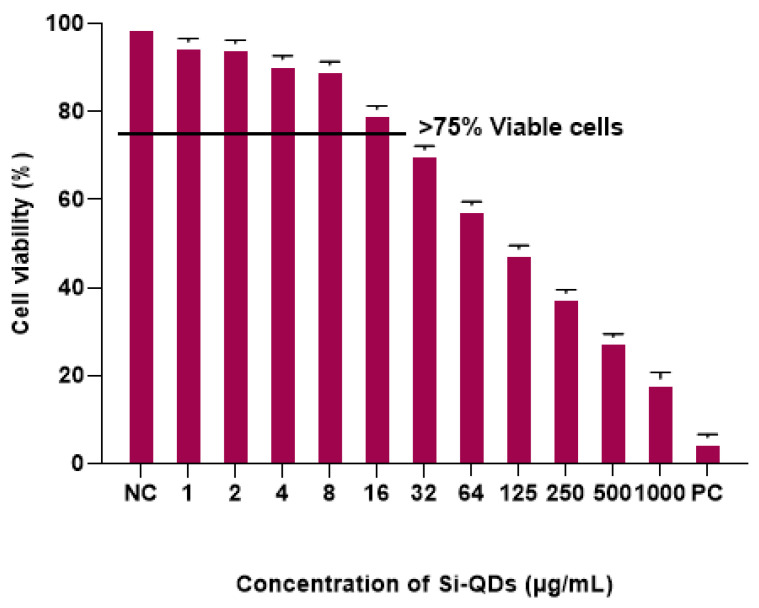
MTT assay: Cell viability (%) of human corneal epithelial cells (HCECs) after exposure to different concentrations of Si-QDs. The horizontal line indicates a cut-off cell viability of more than 75%. Negative control, NC positive control (PC).

Figure 3 shows the percentage viability of HCECs following exposure to different concentrations of Si-QDs. Si-QDs showed an average cell viability of >95% at 1 μg/mL and reduced to 5% at 250 μg/mL, compared to the positive control. Si-QDs exhibited no significant reduction in cell viability at 16 μg/mL and below, relative to the negative control (*p* = 0.15).

### 2.3. In Vitro Fluorescence Imaging of TheraTears^®^ with Si-QDs

**Figure 4:**
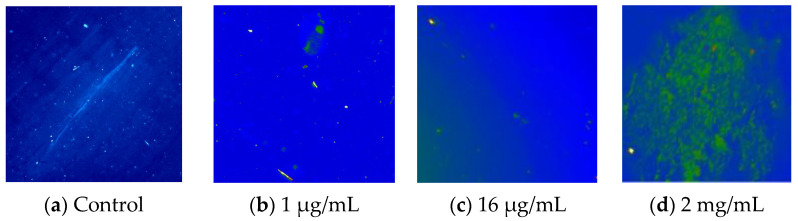
Fluorescence emissions of Sc-Si-QDs in TheraTears^®^ at different concentrations. NC = negative control (TheraTears^®^ alone).

The control solution (TheraTears^®^), devoid of Si-QDs, exhibited no detectable fluorescence signal (Figure 4a). Si-QDs emitted green fluorescence at all given concentrations with scattered fluorescence emission at 16 μg/mL and above. However, Si-QDs appeared to be aggregated at higher concentrations resulting in dispersed fluorescence (Figure 4d).

**Figure 5:**
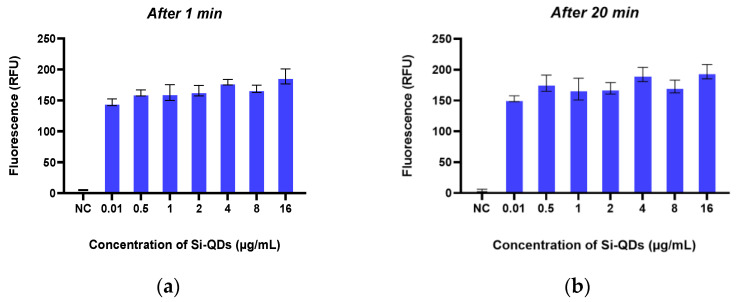
The fluorescence emission of the Sc-Si-QDs at two different time points: after (**a**) 1 min and (**b**) 20 min. RFU: Relative Fluorescence Unit.

Figure 5 illustrates the emission of green fluorescence from Si-QDs at 1 min (Figure 5a) and 20 min (Figure 5b) following their addition to TheraTears^®^. The average fluorescence intensity was 183 RFU at 1 min and 176 RFU at 20 min. The fluorescence intensity of Si-QDs was stable over the 20 min observation time (*p* < 0.05).

**Figure 6:**
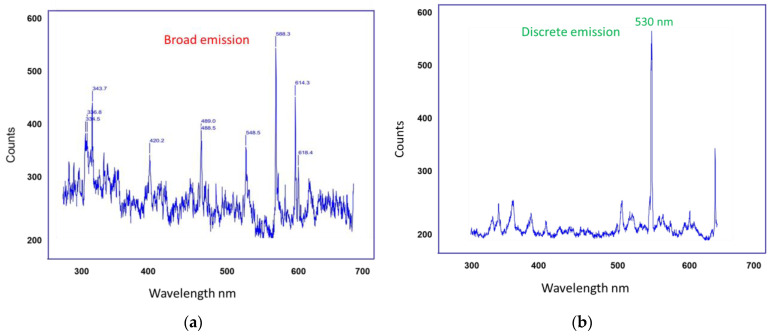
Signal detection from (**a**) Control TheraTears and (**b**) TheraTears + Si-QDs.

Figure 6 illustrates the detection of fluorescence emission wavelength from artificial tears and Si-QDs using SOLIS software. Si-QDs-free TheraTears showed a broader visible light emission (300–700 nm), while Si-QDs emitted at a discrete peak of 530 nm.

### 2.4. Solubility of Si-QDs

**Figure 7:**
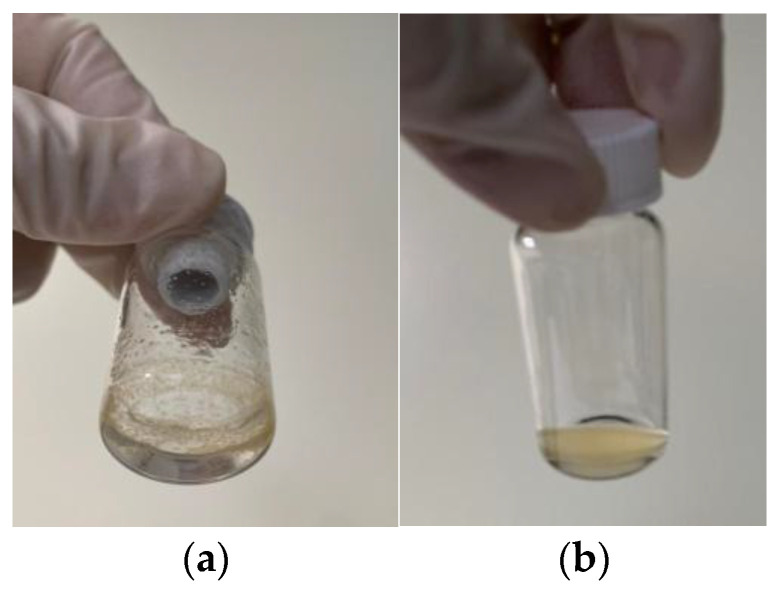
The solubility of Si-QDs in (**a**) MilliQ water and (**b**) Chloroform.

Figure 7 illustrates the solubility of Si-QDs in MilliQ water and Chloroform. In MilliQ water, Si-QDs exhibited minimum solubility as particles aggregated and adhered to the glass walls, and remained at the top surface of the solution. In contrast, Si-QDs showed higher solubility in Chloroform, forming a homogeneous solution.

## 3. Discussion

DED is among the most widespread ocular diseases globally, affecting the quality of vision and daily life [6]. The tear film is essential for developing treatment modalities for DED; however, fundamental knowledge about dynamics remains incomplete [3,5]. QDs provide a novel approach to studying the tear film dynamics [12,22] and an alternative to traditional diagnostic dyes and imaging techniques due to their fluorescence properties and surface functionalisation [13]. The current study demonstrated the optimal size and investigated the fluorescence emission and cytotoxicity of hydrophobic Si-QDs, which could potentially be used as a bioimaging agent to study tear film lipid layer dynamics.

Hydrophobic Si-QDs were synthesised in an aqueous phase [30]. To achieve the quantum yield effect ideal for bright fluorescence emission, a strong reducing agent such as LiAlH4 was used to generate small size particles [31]. This size allows particles to absorb light within a broad spectrum while emitting a narrow wavelength of light [28]. In addition, the surface of Si-QDs was modified with the hexane group to be hydrophobic, making Si-QDs suitable for specific labelling of the tear film’s lipid layer [12]. As visible light is commonly employed in ophthalmic imaging devices for safety purposes, it is important to use a light source to excite QDs while delivering a discrete emission [12].

Si-QDs have strong resistance to photobleaching, a major drawback of the currently used organic fluorescent dyes [30]. This characteristic is specifically important in the tear film imaging, which requires prolonged imaging [12]. Si-QDs exhibited stable fluorescence emission at all given concentrations for 20 min, consistent with prior studies [22,28,30,32]. In addition, Si-QDs emit a suitable wavelength of light, which is desirable for specific bioimaging. Therefore, it may be feasible to dispense hydrophobic Si-QDs into the lipid component of the tear film and capture their dynamics using a fluorescence emission signal. In contrast, it is difficult to monitor individual layers of tear film using fluorescein for a more extended period as it destabilises the tear film during examination [33]. Fluorescein cannot differentiate between aqueous-deficient and evaporative dry eyes [34]. QDs could label specifically the tear film lipid layer and help develop a targeted drug delivery system. Nonetheless, the chance of reduced fluorescence in biological environments still remains; hence, studies on in vivo imaging models are recommended for further optimisation of Si-QDs.

In contrast to hydrophilic Si-QDs, hydrophobic Si-QDs aggregated at certain concentrations, as previously reported [35]. This aggregation likelyresulted from the hydrophobic nature of Si-QDs, which was not sufficiently accommodated by the surfactants present in artificial tear formulations [36]. Under specific conditions, hydrophobic QDs with minimal cosolvents, such as chloroform and hexane, can penetrate membranes non-invasively, suggesting the potential for novel nanoprobes [37]. Therefore, hydrophobic QDs could be solubilised with cosolvents to image tear film lipid layers without aggregation. Polymer encapsulation of hydrophobic particles facilitates solubility in aqueous environments, making QDs suitable for bioimaging [38]. This encapsulation enhances the stability of quantum dots in physiological conditions without compromising their optical properties [38]. Another option is surface functionalisation with polyethylene glycol to increase the solubility of Si-QDs and their biocompatibility in an aqueous environment [39].

One prominent advantage of Si-QDs is their biocompatibility compared to traditional QDs, which often constitute toxic elements such as cadmium and lead [25,27,40,41]. Therefore, this biocompatibility enhances the potential of Si-QDs for use in tear film bioimaging, where direct contact with biological tissue is required [23]. In this study, Si-QDs did not significantly reduce the cell viability of HCECs at 16 µg/mL and below. A concentration-dependent reduction in cell viability was seen at concentrations beyond 16 µg/mL. Molybdenum sulfide QDs exhibited a cell viability of >77% at 250 µg/mL and a dose-dependent reduction in cell viability of Hela cells at higher concentrations [42]. Similarly, copper and carbon hydrophobic QDs also did not exhibit cytotoxic effects on mouse fibroblast cells [43,44]. Cadmium-based QDs have been shown to induce significant cytotoxic effects due to the release of reactive oxygen species [45]. Limited studies indicate no toxicity associated with Si-QDs [23,25], while others showed higher toxicity even with small-sized particles (<6 nm) [46]. No cytotoxic effect was observed with large 50 nm QDs [47]. Hydrophobic QDs show significant potential for ocular applications, but their cytotoxicity varies depending on composition, surface modification, and concentration. Gaining a deeper understanding of these factors is essential for developing safer and more efficient quantum dot-based technologies.

## 4. Materials and Methods

### 4.1. Synthesis and Characterisation of Hydrophobic Sc-Si-QDs

Si-QDs doped with scandium were synthesised in a solution phase [29]. Solution phase synthesis uses surfactants to interact with nanoparticles and limit their growth to reach the optimal size [48]. This synthesis process was conducted under argon atmosphere in a glove box with oxygen levels below 10 ppm and surface functionalisation with Zinc Sulphide [49] to prevent the oxidation of silicon in a biological environment [48]. Therefore, tetraoctyl ammonium bromide in toluene is used. QDs were analysed for their size and photoluminescence by TEM (Olympus Life Science, Notting Hill, VIC, Australia) and spectrofluorophotometer (RF-5301PC Shimadzu, Rydalmere, NSW, Australia). TEM images were taken at an acceleration voltage of 200 kV.

**Figure 8:**
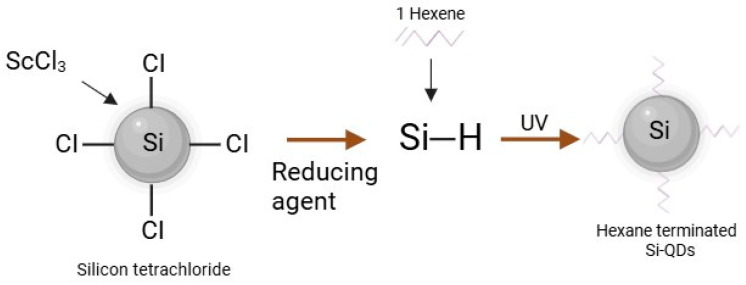
The schematic representation of hydrophobic Si-QDs.

Briefly, as shown in Figure 8 Si-QDs were doped with four scandium atoms per QDs molecule by adding 0.5 g of tetraoctylammonium bromide and 0.026 mmol of ScCl_3_ to a Schlenk tube. The Schenk tube underwent three evaluation cycles along the Schlenk line, followed by 5 min of Nitrogen purging per cycle. A total of 50 mL of anhydrous toluene was added, stirring the solution for 24 h. Silicon tetrachloride was added, and the solution was again stirred for an hour. Lithium aluminium hydride (reducing agent) was added, allowing the solution to react for 3 h. This resulted in hydride Si-QDs doped with Sc. Hydrophobic quantum dots were created by modifying silicon hydrogen bonds by adding 0.1 mol hexachloroplatinic acid in isopropyl alcohol and 1-heptene [48]. The samples for Transmission Electron Microscopy (TEM) were prepared by drop-casting doped Si-QDs in 0.5–1.0 mL of hexane onto carbon-coated copper grids. The emission spectrum is used to obtain the most significant emission wavelengths.

### 4.2. MTT Assay

Human Corneal Epithelial Cells (HCECs) were cultured in Dulbecco’s Modified Eagle Medium (DMEM: Thermo Fisher Scientific, Sydney, Australia) supplemented with 10% Fetal Bovine Medium, 2 ng/mL human recombinant epidermal growth factor, and 1% ITS at 37 °C with 5% CO_2_. The MTT assay was used to evaluate the cytotoxicity of hydrophobic Si-QDs against HCECs after 24 h of exposure [27]. This assay is based on reducing the 3(4,5-dimethylthiazol-2-yl)-2,5-diphenyl tetrazolium bromide (MTT) to dark blue formazan by viable cells in a 96-well plate, incubated at 37 °C in a 5% humidified CO_2_ chamber. HCECs were exposed to different concentrations (2000 µg/mL, 1000 µg/mL, 500 µg/mL, 250 µg/mL, 125 µg/mL, 62.6 µg/mL, 32 µg/mL, 16 µg/mL, 8 µg/mL, 4 µg/mL, 2 µg/mL, 1 µg/mL) of Si-QDs in triplicates for 24 h. DMSO and DMEM were used as positive and negative controls, respectively. After incubation, 100 µL of 5 mg MTT was added to each well containing HCECs and three blank wells, and the plate was incubated for 2–4 h until purple precipitates appeared. The supernatants were removed, and 100 µL of DMSO was added to solubilise the MTT. The absorbance was measured at 570 nm using a spectrophotometer (BMG LABTECH, Ortenberg, Germany). The percentage viability was calculated using absorbance values and plotted against concentrations of Si-QDs. The non-toxic concentrations were further analysed for fluorescence emission.

### 4.3. In Vitro Imaging of Si-QDs

This study used an optimised imaging system based on a previously published procedure to detect fluorescence from Si-QDs [50]. The fluorescence emission of the hydrophobic Si-QDs was detected by mixing a 10 µL aliquot of non-toxic concentrations of Si-QDs (16 µg/mL, 8 µg/mL, 4 µg/mL, 2 µg/mL, 1 µg/mL) with artificial tears (TheraTears^®^, Akorn, Inc., Ann Arbor, MI, USA). TheraTears^®^, a balanced electrolyte formula, was used because it closely mimics the physiology of the tear film. The ultimate goal of Si-QDs is to image the tear film; therefore, optimising their fluorescence in an environment closely resembling the tear film is crucial. In addition, in vitro imaging can provide accurate results without the risk of degradation caused by toxic substances in organic dyes [22]. A high-resolution sCMOS camera (Zyla 5.5 megapixel, Andor Oxford Instruments Group, Belfast, Northern Ireland) attached to a slit lamp biomicroscope (Carl Zeiss, Dublin, CA, USA) was used for in vitro imaging of Si-QDs. Images were taken at a frame rate of 25 per second using Slit Lamp (30×) at time intervals of 1 min, 5 min, 10 min, 15 min, and 20 min. The built-in excitation filter of the slit lamp biomicroscope was used while the emission filter (MF530 FITC Emission Filter; Thorlabs Inc., Newton, New Jersey, USA) was placed in front of the slit lamp objective lens to facilitate discrete fluorescence emission from Si-QDs. The background signal was subtracted using SOLIS software. Three repeated single scans were taken using an exposure time of 1.99 s. A clear microscope slide was used as a negative control.

### 4.4. Assessment of Si-QDs Solubility

The solubility of Si-QDs has been evaluated by their dispersion in aqueous and organic solvents. Si-QDs were added to the MilliQ water and chloroform to achieve the safest concentration of 16 µg/mL. The solution was then vortexed (Vortex Genie 2-Mixer 230 V, 50 Hz) at 1000 RMP/min for 1 min to allow the particles to dissolve. Finally, the resulting solution was observed for its solubility in a solvent.

## 5. Conclusions

Hydrophobic Si-QDs demonstrated optimal size, biocompatibility, and discrete fluorescence emission, suggesting their potential application in imaging the tear film lipid layer. However, unlike hydrophilic Si-QDs, hydrophobic Si-QDs may aggregate on the ocular surface due to nonpolar functional groups and solvents. Based on the results, surface functionalisation and the use of cosolvent are recommended to enhance their solubility using cosolvent. Future research should also involve preclinical ex vivo and in vivo cytotoxic evaluations for further optimisation.

## Data Availability

The raw data supporting the conclusions of this article will be made available by the corresponding author upon request.
